# Evolving Treatment Landscape of *HER2*-mutant Non-Small Cell Lung Cancer: Trastuzumab Deruxtecan and Beyond

**DOI:** 10.3390/cancers15041286

**Published:** 2023-02-17

**Authors:** Ioannis A. Vathiotis, Dimitrios Bafaloukos, Konstantinos N. Syrigos, George Samonis

**Affiliations:** 1 Third Department of Internal Medicine, Sotiria General Hospital for Chest Diseases, School of Medicine, National and Kapodistrian University of Athens, 11527 Athens, Greece; 2 First Oncology Department, Metropolitan Hospital, 18547 Athens, Greece

**Keywords:** NSCLC, HER2, trastuzumab

## Abstract

**Simple Summary:**

By coupling the potency of cytotoxic chemotherapy with the selectivity of targeted therapy, antibody–drug conjugates represent a unique and rapidly growing class of antitumor agents. In this review, we aim to outline the unanswered questions that have emerged after the approval of trastuzumab deruxtecan in *HER2*-mutant non-small cell lung cancer. We also summarize data on novel therapies that are currently being investigated in the same patient population.

**Abstract:**

Successful targeting of *HER2*-activating mutations in DESTINY-Lung02 phase II study has led to the approval of the antibody–drug conjugate (ADC) trastuzumab deruxtecan (T-DXd) as second-line treatment in patients with non-small cell lung cancer (NSCLC). Despite the impressive results, several matters need to be addressed, including the clinical activity of T-DXd in patients with disease in the central nervous system as well as the role of T-DXd in the context of HER2 overexpression. Additionally, data regarding novel agents used to target HER2 continue to accumulate. This review highlights the challenges and unanswered questions that have emerged after the approval of T-DXd in patients with *HER2*-mutant NSCLC.

## 1. Introduction

On 11 August 2022, the Food and Drug Administration (FDA) gave accelerated approval to trastuzumab deruxtecan (T-DXd) for adults with previously treated, unresectable or metastatic non-small cell lung cancer (NSCLC) harboring activating mutations in the gene encoding human epidermal growth factor receptor 2 (HER2), based on an interim efficacy analysis of DESTINY-Lung02 phase II clinical trial [1]. In the latter, a total of 80 patients with *HER2*-mutant NSCLC who had received at least one prior line of treatment, mostly platinum-based chemotherapy, were randomized in a 2:1 ratio to receive T-DXd at a dose of 5.4 mg/kg or 6.4 mg/kg, respectively. Patients receiving T-DXd at the 5.4 mg/kg dose level demonstrated a numerically superior objective response rate (ORR; 57.7%), confirmed by a blinded independent central review (BICR), along with a more favorable toxicity profile; any-grade adjudicated drug-related interstitial lung disease (ILD) was seen in 5.9% and 14.0% of patients receiving T-DXd at 5.4 mg/kg and 6.4 mg/kg, respectively.

T-DXd is an antibody–drug conjugate (ADC) consisting of a humanized monoclonal IgG1 antibody against HER2 (trastuzumab), linked to a topoisomerase I inhibitor (deruxtecan) payload through a cleavable tetrapeptide-based linker [2]. Compared to other drugs of the same class, this system incorporates a high drug-to-antibody ratio (DAR; 8 molecules of topoisomerase inhibitor per antibody), with high stability in plasma, exhibiting predictable pharmacokinetics.

Since the first approval of a targeted therapy in patients with NSCLC (erlotinib in epidermal growth factor receptor [EGFR]-positive disease) in 2013, molecular testing has evolved into an essential component of patient care with the National Comprehensive Cancer Network currently advising for broad molecular profiling to assess nine predictive biomarkers, at least, at baseline [3]. Among these, activating *HER2* mutations are identified in 1–3% and 1.4–6.7% of NSCLC patients of European/American and Asian descent, respectively; in-frame insertion mutations occurring in exon 20 represent the most common subtype, seen in approximately 48% of the cases [4,5,6,7]. In the vast majority, de novo *HER2* mutations are mutually exclusive with other driver alterations. They are most commonly present in females and never-smokers with pure adenocarcinoma histology or an adenocarcinoma component. Interestingly, NSCLC patients with *HER2* mutations tend to exhibit a more aggressive disease phenotype with increased incidence of brain metastases during treatment; this association is significantly higher in individuals harboring the exon 20 A775_G776insYVMA insertion compared to non-YVMA cases [8,9]. Activating *HER2* mutations have been shown to enhance receptor internalization in preclinical models, providing a potential explanation for the observed clinical efficacy of T-DXd regardless of the quantity of HER2 protein expression [10,11]. Previous studies have also confirmed the clinical efficacy of T-DXd across *HER2* mutation subtypes [12].

In the subsequent sections, we will review the evidence on current challenges and unanswered questions regarding the implementation of T-DXd in the treatment algorithm of *HER2*-mutant NSCLC. Furthermore, we will summarize available data on novel strategies to target HER2 in patients with NSCLC.

## 2. Central Nervous System Involvement

Patients with HER2-driven tumors have a propensity to develop central nervous system (CNS) disease, which bears a negative impact on overall survival (OS) as well as quality of life [13]. Accumulating data support the clinical efficacy of T-DXd in breast cancer patients with CNS involvement, which may provide an opportunity for transition to tumor types with fewer data on the intracranial efficacy of T-DXd, including NSCLC. In the phase III DESTINY-Breast03 clinical trial, T-DXd conferred a significant improvement in progression-free survival (PFS), ORR and intracranial response rate compared with T-DM1 in 82 patients with HER2-positive breast cancer and stable brain metastases at baseline; the overall intracranial response rate was 63.8% for T-DXd compared with 33.3% for T-DM1 with the percentage of patients who achieved a complete intracranial response being 27.8% and 2.8%, respectively [14]. Notably, patients treated with T-DXd had a median PFS of 15 months. In addition, a recent subgroup analysis of DESTINY-Breast01 confirmed the durable clinical effect of T-DXd in patients with stable brain metastases [15]. 

The CNS activity of T-DXd has been proven in patient-derived xenograft models of HER2-positive as well as HER2-low breast cancer brain metastases [16]. The DEBBRAH phase II study assessed the efficacy of T-DXd in pretreated patients with either HER2-positive or HER2-low breast cancer and involvement of the CNS [17]. The intracranial ORR in patients with asymptomatic untreated brain metastases and those with brain metastases progressing after local therapy was 50% and 44.4%, respectively. In the TUXEDO-1 phase II study, treatment with T-DXd resulted in an intracranial response rate of 73.3% (by RANO-BM; 11/15 patients) and a median PFS of 14 months in patients with HER2-positive breast cancer and newly diagnosed or progressive brain metastases; the response was 100% in patients with de novo brain metastases and 66.7% in patients with brain metastases that had progressed after previous local therapy [18]. 

## 3. HER2-Amplified/Overexpressing Disease

Following the successful implementation of HER2-directed therapies in patients with breast cancer, strategies targeting HER2 have been investigated in other tumor types, including NSCLC [19]. Although the addition of trastuzumab did not confer survival benefit in patients with HER2-amplified/overexpressing NSCLC, promising results were seen with trastuzumab-emtansine (T-DM1), with responses correlating with the levels of HER2 protein expression by immunohistochemistry (IHC), but the latter was still regarded as a rather insufficient predictive biomarker [20,21,22]. In the case of T-DXd, the first signs of antitumor activity were documented by Tsurutani et al., in a mixed population of heavily pretreated HER2-expressing or *HER2*-mutant solid tumors [23]. Subsequently, the efficacy of T-DXd in HER2-overexpressing NSCLC was evaluated in a DESTINY-Lung01 phase II clinical trial [11]. In this study, a cohort of 49 patients with HER2 IHC 2+ (*n* = 39) or 3+ (*n* = 10) received T-DXd at 6.4 mg/kg after disease progression to prior anticancer therapy (median number of prior regimens was three). Confirmed ORR was 24.5%, including 25.6% in patients with IHC 2+ and 20.0% in patients with IHC 3+; the estimated median PFS reached 5.4 months. As a result, an additional cohort has opened to assess matters of safety and efficacy of T-DXd given at 5.4 mg/kg in the same population.

Based on the results of DESTINY-Breast04, the benefit from T-DXd administration has been extended to patients with HER2-low breast cancer, defined as IHC 2+ with negative results on in situ hybridization (ISH) or IHC 1+ [24]. Unlike other anti-HER2 agents, HER2 overexpression may not be required for the efficacy of T-DXd; as a matter of fact, T-DXd has demonstrated signs of antitumor activity across a full range of HER2 expression, essentially redefining HER2-positive disease [25]. Given the preanalytical as well as analytical factors that may explain, to some degree, patient misclassification when HER2 testing is performed by IHC, several novel quantitative assays with the potential to enhance the sensitivity of HER2 assessment have emerged [26,27,28]. As suggested by Tarantino et al., successful targeting of HER2 in nonamplified tumors has the potential to alter the current treatment landscape in several ways, including mitigation of oncogene dependency (as novel ADCs may exert their antitumor activity regardless of tumor cell dependency on the HER2 pathway), higher DAR/alternative payload mechanisms of action, and the use of modern cleavable linkers that have the potential to facilitate the “bystander effect”. Recently, the antitumor efficacy of T-DXd in HER2-low disease was also observed in gastric or gastroesophageal junction adenocarcinoma, with confirmed ORR equal to 26.3% and 9.5% in patients with IHC 2+/ISH-negative and IHC 1+ tumors, respectively [29].

## 4. Other ADCs

ADCs represent an innovative class of anticancer drugs that combine the selectivity of targeted therapies with the potency of cytotoxic chemotherapy. HER2 has long represented an attractive target for ADCs in NSCLC (Table 1) [30,31]. Composed of the anti-HER2 antibody trastuzumab linked to the microtubule inhibitor emtansine (DM1) via a non-cleavable linker, T-DM1 was the first ADC to be approved for the treatment of HER2-positive breast cancer and the first one to be evaluated in patients with NSCLC; T-DM1 has a DAR of 3.5 [30,32]. In a phase II basket trial, treatment with T-DM1 resulted in an ORR of 44.4%, meeting the primary study endpoint, and a median PFS of 5 months in heavily pretreated patients with *HER2*-mutant NSCLC [21]. Subsequently, Li et al. enrolled more patients to confirm the efficacy (ORR equal to 51%) and tolerable toxicity profile of T-DM1, with only one incidence of grade 3 or higher treatment-related adverse events (TRAEs) [10]. Lately, treatment with T-DM1 showed slightly inferior results in the Japanese population [33].

A166 is a novel ADC consisting of trastuzumab and monomethyl auristatin F derivative duostatin-5, linked with a cleavable linker. A166 exhibited clinical efficacy in 35 heavily pretreated patients with HER2 amplified/overexpressing advanced solid tumors [34]. The ORR of A166 was 36% with responses seen only at the 3.6 mg/kg and the 4.8 mg/kg dose level; ophthalmic toxicities of any grade, including keratitis, dry eye, blurred vision, etc., appeared in more than 80% of study participants at efficacious dose levels. In a second phase I study, A166 demonstrated a manageable toxicity profile with grade 3 or higher treatment-related adverse events (TRAEs) recorded in 18 out of 57 study participants (31.6%) [35]. Moreover, it showed high stability in plasma and promising antitumor activity. Among 36 HER2-positive breast cancer patients eligible for efficacy assessment, the best ORR was 59.1% (13/22) and 71.4% (10/14) at the 4.8 mg/kg and 6.0 mg/kg dose level, respectively; the median PFS was not reached at the time of data cutoff.

XMT-1522 is an ADC that consists of a human IgG1 anti-HER2 monoclonal antibody (HT-19) that binds to a distinct epitope of HER2, compared with trastuzumab, conjugated to auristatin F-hydroxypropylamide via a cleavable linker; this ADC has a DAR of 12. Preclinical evidence shows increased potency of XMT-1522 compared with T-DM1, as well as antitumor activity in the case of primary or acquired resistance to T-DM1 in vitro [36]. Preliminary results of a phase I study indicated that XMT-1522 was well tolerated up to the dose of 21.3 mg/m^2^ (all TRAEs were grade 1 and 2, most commonly elevated liver function tests and fatigue) and demonstrated early signs of antitumor activity [37]. 

ARX788 is a next-generation, site-specific ADC, composed of an anti-HER2 monoclonal antibody and the microtubulin inhibitor amberstatin (AS269; DAR, 1.9), that has already produced promising preliminary results in patients with HER2-positive metastatic breast cancer and is currently being evaluated in multiple solid tumors, including breast and gastric/gastroesophageal junction carcinoma (NCT03255070) [38,39]. Finally, NCT04235101 is investigating matters of safety for the combination of SYD985 (trastuzumab duocarmazine) with niraparib in patients with solid tumors.

## 5. New-Generation Tyrosine Kinase Inhibitors

Given the small size, flexible structure and covalent binding properties, novel tyrosine kinase inhibitors (TKIs; i.e., poziotinib) have demonstrated increased potency compared with the previous generation. Results from two phase II studies were highly concordant for poziotinib, indicating modest antitumor activity in previously treated patients with NSCLC and *HER2* exon 20 insertion mutations, with an ORR of about 27% [40,41]. The median PFS was 5.5 months and the median overall survival (OS) reached 15 months. It should be noted that these response rates are well below compared with TKIs used in *EGFR*-driven NSCLC. The most common grade 3 or higher TRAE was rash, seen in nearly half of the patients, followed by diarrhea and stomatitis. Importantly, dose reductions were required in 76.7% of study participants, while treatment discontinuation occurred in 13.3%. On the basis of increased toxicity as well as lack of phase III data, the Food and Drug Administration (FDA) denied approval of poziotinib for this indication. 

Showing activity against secondary C805S mutations, which represent the most common mechanism of acquired resistance to poziotinib, pyrotinib has also been evaluated in *HER2*-mutant NSCLC. In a phase II study, pyrotinib exhibited more promising antitumor activity (ORR was 30% with efficacy seen across *HER2* mutation subtypes, median PFS was 6.9 months and median OS was 14.4 months), along with a rather acceptable toxicity profile (grade 3 or higher TRAEs were documented in 28.3% of patients, with the most common being diarrhea) [42]. 

Further attempts to target exon 20 insertions have included mobocertinib (TAK-788), a novel EGFR/HER2 TKI with high selectivity (low *HER2* ex20ins IC50/wildtype *EGFR* IC50 ratio), increased efficacy against the G776 > VC subtype, and potential for synergy with T-DM1, with the latter being largely driven by M1 macrophage infiltration and CD4+ T cell activation [43]. Mobocertinib has already been FDA-approved in patients with NSCLC harboring *EGFR* exon 20 insertions (NCT02716116), resulting in an expansion cohort of patients with *HER2* exon 20 insertions [44,45].

The high homology among ERBB family members has made it problematic to target HER2 while sparing other tyrosine kinases. Lack of selectivity has, thus, resulted in decreased potency and dose-limiting toxicity, hampering most efforts to target this disease in the clinic [46]. To circumvent the on-target toxicity caused by wild-type EGFR inhibition, tarloxitinib is a prodrug that can be converted to its active form, tarloxitinib-E, in the hypoxic tumor microenvironment [47]. Tarloxitinib-E has been shown to directly inhibit the phosphorylation and activation of EGFR, HER2 and HER2/HER3 heterodimers in vitro, resulting in tumor regression in vivo. Furthermore, tarloxitinib displays an IC50 about 180 times higher for wild type HER2 than the active metabolite, demonstrating a wide therapeutic index [48]. Importantly, secondary C805S *HER2* mutations, as well as *HER3* overexpression, have been identified as mechanisms of acquired resistance to tarloxotinib-E. In cohort B of the RAIN-701 phase I study (NCT03805841), tarloxitinib-E showed antitumor activity with an ORR of 22% and acceptable toxicity profile; the most common grade 3 toxicity was QTc interval prolongation, which appeared in 34.8% of patients [49].

Further attempts to address this particular issue led by Neumüller and colleagues have resulted in the development of selective HER2 inhibitors with a large therapeutic window (>50-fold for HER2 inhibition compared to wild-type EGFR); the presence of a hydrogen bond to serine 783 in the back pocket of HER2, which is not formed with the corresponding cysteine 775 in EGFR, establishes high selectivity [50]. Interestingly, selective HER2 YVMA inhibition was shown, which suffices to suppress tumor growth, as inhibition of wild-type EGFR did not appear to confer a meaningful therapeutic effect. JBJ-08–178-01 is a novel TKI effective against *HER2*-activating mutations as well as amplification both in vitro and in vivo [51]. It exhibits increased selectivity in comparison with other TKIs, inhibiting HER2 over wild-type EGFR; moreover, it has been shown to reduce the levels of HER2 by inducing proteasomal degradation of the receptor. Finally, BI 1810631 is a HER2-selective TKI that engages both wild-type and mutated HER2 receptors but spares EGFR signaling and is currently being evaluated in the context of a phase I study (NCT04886804) [52]. 

## 6. Bispecific Antibodies

Bispecific antibodies (bsAbs) are molecules designed to recognize two different epitopes or antigens [53]. Since the first mention of the concept in 1960, dual targeting enabled by bsAbs has sparked research interest leading to novel features and functionalities that cannot be accomplished with a simple mixture of the reference clones; physical linkage of two binding sites may in fact create temporal or spatial dependency [54]. As a result, the bsAb landscape is rapidly expanding, providing opportunities for enhanced epitope targeting and drug development.

As such, zenocutuzumab (MCLA-128) is a humanized IgG1 bsAb that is designed to target the extracellular domains of HER2 and HER3 using a unique “dock and block” mechanism [55]. The HER2-targeting arm binds to HER2 on the cell surface and in turn sets the HER3-targeting arm in place to prevent neuregulin 1 (NRG1) binding to HER3; the latter has been shown to induce a conformational change that is required for HER2:HER3 heterodimerization and downstream signaling. In addition, IgG1 glycosylation results in enhanced antibody-dependent cellular cytotoxicity (ADCC). Treatment with zenocutuzumab has been shown to decrease phosphorylation of HER2, HER3, EGFR, and HER4 and reduce NRG1 fusion-dependent oncogenic signaling, thereby suppressing tumor growth in preclinical models [56]. Moreover, zenocutuzumab has demonstrated promising antitumor activity leading to durable responses in heavily pretreated patients with *NRG1* fusion-positive disease and a tolerable toxicity profile [57]. Based on these, it is currently being investigated in the context of a phase I/II study (NCT02912949). 

## 7. Immune Checkpoint Inhibitors

Checkpoint inhibitor-based immunotherapy targeting the programmed cell death protein 1 (PD-1)/programmed death-ligand 1 (PD-L1) axis has acquired a central role in the management of patients with locally advanced or metastatic driver-negative NSCLC. However, the presence of driver alterations has oftentimes been associated with a poor response to immunotherapy. In particular, *EGFR*-mutant tumors harbor a low tumor mutational burden (TMB) and a “cold” tumor microenvironment, characterized by restricted T-cell clonality, and these features appear culpable to lack of benefit from PD-(L)1 inhibitors [58,59,60,61,62]. Moreover, the predictive value of PD-L1 expression in epidermal growth factor receptor (EGFR)-positive disease has not been established yet; preclinical data have suggested that PD-L1 expression may reflect EGFR signaling rather than T-cell activity. However, this may not be the case for every single actionable alteration in NSCLC [63]. 

The presence of activating *HER2* mutations has been associated with low prevalence of PD-L1 positivity (47.6%) as well as low TMB (median <3 mut/Mb) [63]. Although multiple studies have investigated the role of PD-(L)1 inhibition in patients with NSCLC and activating *HER2* mutations, current evidence remains retrospective in nature (Table 2). Lai et al. documented an ORR of 11.5% and median PFS of 1.9 months among 26 such patients treated with single-agent PD-(L)1 inhibitors [64]. Similar results were obtained by the IMMUNOTARGET registry, where 29 patients that received anti-PD-(L)1 therapy beyond the first line of treatment demonstrated an ORR of 7.4% and median PFS of 2.5 months [65]. Although the French Lung Cancer Group (GFPC) noted objective responses in six out of 23 (27.3%) patients with *HER2*-mutant tumors treated with PD-1/PD-L1 axis inhibitors, the median PFS did not exceed 2.2 months [66]. Negrao et al. reported on two different cohorts of unselected NSCLC patients with actionable alterations including activating *HER2* mutations (MD Anderson Cancer Center [MDACC], *n* = 15; Flatiron Health-Foundation Medicine Clinico-Genomic Database [CGDB], *n* = 28) [63]. Again, patients with *HER2*-mutant tumors demonstrated poor outcomes with median PFS of 1.9 months and 3.0 months for the MDACC and CGDB cohort, respectively. Still, NSCLC patients harboring activating mutations in *HER2* exhibit significantly better outcomes compared with those harboring sensitizing *EGFR* mutations, except for *EGFR* exon 20 insertions [67].

Given the limited benefit seen with checkpoint inhibitor monotherapy, recent efforts have focused on chemoimmunotherapy combinations. As such, among 13 NSCLC patients with *HER2* exon 20 insertions the ORR was 30.8% with the combination of chemotherapy and immunotherapy [68]. In addition, Saalfeld et al. reported an ORR of 52.4% and median PFS of 6 months in treatment-naive patients that received pembrolizumab-based chemoimmunotherapy, as opposed to 16.1% and 4 months, respectively, for patients that received immune checkpoint inhibitor monotherapy beyond the first line of treatment [69]. Real-world data from the POLISH study indicated that first-line chemoimmunotherapy may achieve comparable results to chemotherapy plus angiogenesis inhibition with bevacizumab in the Chinese population; among 46 patients with actionable *HER2* alterations, chemoimmunotherapy combinations achieved an ORR of 29% and median PFS of 5.2 months [70]. 

Currently, several clinical trials are testing combinations of PD-(L)1 inhibitors with anti-HER2 agents in patients with locally advanced or metastatic NSCLC. As such, NCT04042701 is a phase Ib study that will assess matters of safety and efficacy for the combination of T-DXd and pembrolizumab in HER2-expressing or *HER2*-mutant disease. Moreover, in the NCT04144569 phase II study, 30 patients with *HER2* insertion mutation-positive advanced NSCLC will receive a PD-1 inhibitor plus pyrotinib after failure of first-line chemotherapy. 

## 8. Conclusions

HER2 alterations represent important oncogenic drivers in NSCLC. During the past couple of years, progress has been made toward defining *HER2*-driven disease and determining the benefit from different classes of agents targeting HER2. Currently, platinum-based chemotherapy with/without immunotherapy is the preferred first-line treatment in patients with advanced or metastatic *HER2*-mutant NSCLC; T-DXd has been approved for the same subgroup of patients after receipt of prior systemic therapy. The European Society of Medical Oncology (ESMO) recommends upfront *HER2* mutation testing (preferentially sequencing of exon 20) as part of a larger routine testing panel using next-generation sequencing (NGS), in patients with unresectable stage III and IV NSCLC who meet two or three of the following criteria, (i) lung adenocarcinoma or adenosquamous carcinoma; (ii) never-smoker status; (iii) female [4]. In addition, testing for *HER2* amplification (by NGS or FISH), as well as HER2 expression (by IHC) can be considered for individuals in clinical studies and, in the case of EGFR TKI resistance, to explore the related resistance mechanisms. 

With more agents in the pipeline, ongoing and future studies will need to provide answers to several questions including the role of T-DXd in first-line treatment (DESTINY-Lung04; NCT05048797), and the potential combination (NCT05048797 and NCT04686305) or sequencing of T-DXd with immunotherapy. In the case of TKIs, an important challenge lies in balancing treatment benefit with treatment-related toxicities that emanate from unselective inhibition of other ERBB family members; the latest efforts to target *HER2*-mutant NSCLC have employed highly selective HER2 inhibitors. Although we are in dire need of mature OS data as well as phase III data, the low incidence of *HER2* mutations hampers the conducting of randomized controlled clinical trials. Finally, the impact of searching for and targeting *HER2* mutations in early disease stages, as is the case in HER2-positive breast cancer, remains yet to be elucidated.

## Figures and Tables

**Table 1:** Clinical trials evaluating antibody–drug conjugates in patients with NSCLC harboring HER2 aberrations. NSCLC, non-small cell lung cancer; HER2, human epidermal growth factor receptor 2; DAR, drug-to-antibody ratio; ORR, objective response rate; PFS, progression-free survival; CI, confidence intervals; OS, overall survival; TRAE, treatment-related adverse event; IHC, immunohistochemistry; FISH, fluorescence in situ hybridization; NE, not evaluable; NA, not available; NR, not reached.

Agent	Antibody; Chemotherapeutic	Study	Phase	*n*	Prior Systemic Therapy	HER2 Positivity Definition	ORR *n* (%)	PFS in Months Median (95% CI)	OS in Months Median (95% CI)	Most Common Grade 3 or Higher TRAE (%)
T-DM1	Trastuzumab; emtansine	Hotta et al. [20].	2	15	Yes	IHC 3+, 2+/FISH+, or exon 20 mutation	1 (6.7)	2.0 (1.4–4.0)	10.9 (4.4–12.0)	Thrombocytopenia (40.0)
T-DM1	Trastuzumab; emtansine	Peters et al. [22].	2	29; 20	Yes	IHC 2+; IHC 3+	0 (0); 4 (20)	2.6 (1.4–2.8); 2.7 (1.4–8.3)	12.2 (3.8–23.3); 15.3 (4.1-NE)	NA
T-DM1	Trastuzumab; emtansine	Li et al. [10]	2	49	Yes	Activating mutation or amplification	25 (51.0)	5.0 (3.5–5.9)	NA	Thrombocytopenia (6.0), Anemia (6.0)
T-DM1	Trastuzumab; emtansine	Iwama et al. [32]	2	22	Yes	Exon 20 insertion mutation	8 (38.1)	2.8 (1.4–4.4)	8.1 (3.5–13.2)	Thrombocytopenia (18.2)
T-DXd	Trastuzumab; deruxtecan	Tsurutani et al. [23]	1	18	Yes	IHC 1+, 2+, 3+ or amplification	10 (55.6)	11.3 (5.5–14.1)	NR (17.3-NE)	NA
T-DXd	Trastuzumab; deruxtecan	Li et al. [12].	1	91	Yes	Mutation	50 (54.9)	8.2 (6.0–11.9)	17.8 (13.8–22.1)	Neutropenia (19.0)
T-DXd	Trastuzumab; deruxtecan	Nakagawa et al. [11]	1	49	Yes	IHC 2+, 3+	12 (24.5)	5.4 (2.8–7.0)	NA	Neutropenia (20.4)
T-DXd	Trastuzumab; deruxtecan	Goto et al. [1]	2	52 (5.4 mg/kg); 28 (6.4 mg/kg)	Yes	Mutation	28 (53.8); 12 (42.9)	NA	NA	NA

**Table 2:** Studies evaluating PD-1 axis blockade in patients with NSCLC harboring HER2 aberrations. NSCLC, non-small cell lung cancer; HER2, human epidermal growth factor receptor 2; PD-1, programmed cell death protein 1; IO, immunotherapy; PD-L1 TPS, programmed death-ligand 1 tumor proportion score; ORR, objective response rate; PFS, progression-free survival; CI, confidence intervals; OS, overall survival; NA, not available; NR, not reached.

Study	Design	*n*	Prior Systemic Therapy	IO Regimen	PD-L1 TPS ≥ 50 (%)	ORR *n* (%)	PFS in Months Median (95% CI)	OS in Months Median (95% CI)
Lai et al. [64]	Retrospective	26	NA	NA	NA	3 (11.5)	1.9 (1.5–4.0)	10.4 (5.9–NA)
Mazieres et al. [64]	Retrospective	29	Yes	Monotherapy	0	2 (7.4)	2.1 (1.3–4.7)	21.3 (3.8–28.0)
Guisier et al. [65]	Retrospective	23	Yes	Monotherapy	4.3	6 (27.3)	2.2 (1.7–15.2)	20.4 (9.3–NR)
Negrao et al. [63]	Retrospective	15; 28	NA; NA	Monotherapy; monotherapy	NA; NA	NA; NA	1.9 (1.6–2.1); 3.0 (1.8–NA)	16.8 (3.1–30.6); 10.8 (5.6–NA)
Lau et al. [63]	Retrospective	14	Yes (78.6)	Monotherapy, combination with anti-CTLA-4	3 (23.1)	4 (28.6)	3.6 (1.6–NR)	NA
Tian et al. [63]	Retrospective	13	No (76.9)	Combination with chemotherapy	NA	4 (30.8)	8.0 (5.2–NR)	NA
Saalfeld et al. [63]	Retrospective	61	No (44.3)	Monotherapy, combination with chemotherapy	15.5	11 (52.4), 1st line chemoimmunotherapy; 5 (16.1), >1st line monotherapy	6.0 (6.0–14.0) 1st line chemoimmunotherapy; 4.0 (4.0–6.0), >1st line monotherapy	NR, (NA–NA) 1st line chemoimmunotherapy; 10.0 (6.0–NA), >1st line monotherapy
Yang et al. [63]	Retrospective	46	No	Combination with chemotherapy	6.5	13 (28.9)	5.2 (3.6–6.8)	NR (NA–NA)
